# Taking a Closer Look: The Relationship between Pre-School Domain General Cognition and School Mathematics Achievement When Controlling for Intelligence

**DOI:** 10.3390/jintelligence10030070

**Published:** 2022-09-19

**Authors:** Antje Ehlert, Nadine Poltz, Sabine Quandte, Juliane Kohn, Karin Kucian, Michael Von Aster, Günter Esser

**Affiliations:** 1 Department of Inclusive Education, University of Potsdam, 14476 Potsdam, Germany; 2 Academy of Psychotherapy and Intervention Research Potsdam, 14467 Potsdam, Germany; 3 Center for MR-Research, University Children’s Hospital, 8032 Zurich, Switzerland; 4 Center for Special Educational and Psychological Needs, DRK Kliniken Berlin Westend, 14050 Berlin, Germany

**Keywords:** intellectual ability, intelligence, pre-school, mathematical precursor, mathematical development, school mathematics, longitudinal, numerical skills, working memory, attention

## Abstract

Intelligence, as well as working memory and attention, affect the acquisition of mathematical competencies. This paper aimed to examine the influence of working memory and attention when taking different mathematical skills into account as a function of children’s intellectual ability. Overall, intelligence, working memory, attention and numerical skills were assessed twice in 1868 German pre-school children (t1, t2) and again at 2nd grade (t3). We defined three intellectual ability groups based on the results of intellectual assessment at t1 and t2. Group comparisons revealed significant differences between the three intellectual ability groups. Over time, children with low intellectual ability showed the lowest achievement in domain-general and numerical and mathematical skills compared to children of average intellectual ability. The highest achievement on the aforementioned variables was found for children of high intellectual ability. Additionally, path modelling revealed that, depending on the intellectual ability, different models of varying complexity could be generated. These models differed with regard to the relevance of the predictors (t2) and the future mathematical skills (t3). Causes and conclusions of these findings are discussed.

## 1. Introduction

Learning is the most powerful mechanism of cognitive development. This statement is universally valid for gifted children as well as for less proficient students. High intelligence may only lead to benefits if it is translated into domain-specific knowledge beforehand (Weinert 2001). Knowledge may compensate for a lack of intelligence, whereas high levels of intelligence cannot make up for insufficient knowledge (Schneider et al. 1989). The common understanding is that the combination of domain-general abilities such as intelligence and working memory as well as domain-specific knowledge contributes to academic and professional learning (e.g., Geary et al. 2017; Ferrer and McArdle 2004; Von Aster and Shalev 2007). However, the relative contribution to learning provided by these abilities is a debatable issue, as is the question of whether these contributions change over time or depend on the level of expertise.

### 1.1. The Influence of Domain-General Abilities and Domain-Specific Knowledge on Mathematics Achievement Depending on Developmental Changes and Age

Intelligence influences our abilities on every level of cognitive tasks. Generally, we associate good performance in mathematics with a high intelligence. However, several studies have found that someone with a moderate intelligence level can show great mathematic skills (e.g., Murayama et al. 2012; Saß et al. 2017). In line with this, Rajkumar and Hema (2018) have found that mathematics achievement correlates positively with intelligence. Nonetheless, the majority of assessed undergraduate students (*n* = 310) showed a moderate level of general intelligence. This raises the question of which abilities may predict mathematics achievement.

Domain-general predictors, intelligence and working memory components (Baddeley and Hitch 1974; Baddeley 1986, 2012) are the most commonly studied factors of the development of mathematical competences (e.g., Fuchs et al. 2016; Geary 2011; Lee and Bull 2016). For linguistic competences (Watts et al. 2014), intelligence and working memory abilities are the most consistent factors of short- as well as long-term influences (Friso-van den Bos et al. 2013; Gathercole et al. 2005; Geary 2011; Lee and Bull 2016; Mazzocco and Kover 2007; Siegler et al. 2012; Toll et al. 2011). However, research results disagree on how the influence of domain-general abilities and domain-specific knowledge on mathematical performance may change depending on a learner’s development (e.g., Geary et al. 2017; Ferrer and McArdle 2004; Von Aster and Shalev 2007).

These changes appear to depend on the use of varying domain-general and domain-specific factors, the assessed age groups as well as different empirical analytical methods. For example, intelligence measured two years prior influences academic achievement (Ferrer and McArdle 2004), whereas the intelligence of grade six students, who are merely two years older, cannot predict an increase of academic abilities until ninth grade and beyond the influence of their past abilities (Gustafsson and Undheim 1992). Using autoregressive cross-lagged models, Lee and Bull (2016) found a stable inter-year influence of working memory on mathematics achievement in the following year. In the process, the relevance of previous performance in mathematics increased across all grades. Children with a higher capacity of working memory or updating reached higher achievements in mathematics. Modeling the latent increase shows that higher capacities of working memory or updating in kindergarten predict greater increases for mathematics when averaged across all ages. However, the increase of working memory and updating capacity is invariant across different grades. Neither kindergarteners’ gender nor sex, but rather their socioeconomic status, explain the variance of capacity.

Moreover, the predictive influence of various working memory components at different ages remains unclear. A recent study by Liang et al. (2022, Jun) shows that verbal working memory predicts fifth graders’, but not first graders’, mathematic performance. Nonetheless, the visual-spatial working memory plays a vital role for both age groups. According to McKenzie et al. (2003), children of different ages employ different strategies for simple mental arithmetic tasks: younger children almost exclusively use visual-spatial strategies, whereas older children apply a combination of phonological and visual-spatial strategies. Allen et al. (2020) have found an age-dependent relationship with an increasing influence of visual-spatial components as children get older. Allen et al. (2020) elaborate that for children of school age, the link between working memory and mathematics is essentially positive. However, the type of relationship as well as the cumulative acquisition of mathematic skills vary by age (Li and Geary 2013; Soltanlou et al. 2015; Van de Weijer-Bergsma et al. 2015). As demonstrated by Schneider (2008), Allen et al. (2020) summarize that the age of the assessed children and adolescents is relevant for the expected extent of involvement of the particular components. Additionally, mathematical knowledge is acquired in relation to the individual mathematical domains and their related strategies. Consequently, the patterns of involvement of the different working memory components vary depending on students’ age and mathematical domains (Friso-van den Bos et al. 2013). In a meta-analysis, Friso-van den Bos et al. (2013) have detected a link between working memory and mathematics for 4- to 12-year-olds. Although the correlation between working memory and mathematic abilities is stronger for younger children, the influence of verbal working memory increases with age. The visual-spatial working memory’s relevance has also been identified in other studies: deficits of the visual-spatial component have even proven to be relevant to the development of dyscalculia (Mammarella et al. 2018; Szűcs et al. 2013). However, the relationship between the central executive and mathematics differs. According to Imbo and Vandierendonck (2007), school children draw on resources of their working memory to solve simple arithmetic tasks. This load on their executive working memory resources resulted in worse performance while calculating. Imbo and Vandierendonck (2007) have found that this strain on their central executive leads to similar consequences for recollection abilities of children and adults. Thus, working memory resources are needed to recall information stored in the long-term memory.

Previous research revealed a relationship between the domain-general ability attention and mathematical skills. Shalev et al. (1995) have found that children with dyscalculia show significantly more attention problems than their averagely performing peers. Attention deficit/hyperactivity disorder and mathematics disorders occur comorbidly in school age with a prevalence of 18.1% (Capano et al. 2008). In addition, inconsistent response times and commission errors (incorrectly marked non-target letters) in a continuous attention performance test were found to predict math performance (Lindsay et al. 2001). In particular, visual attention in kindergarten was found to be a good predictor of later mathematical ability. Results of Poltz et al. (2022) show that domain-general cognitive abilities (non-verbal intelligence, visuospatial working memory and visual attention) explain a small but significant proportion of children’s tendencies to spontaneously focus on numerosity.

Likewise, the type of instrument chosen for assessing working memory performance appears to influence the predictive relevance of measurement models. Researchers may distinguish between simple and complex span measures (see Engle 2010) or they may follow requirements of the dual-task paradigm. As span measures are the preferred method when examining children (Allen et al. 2020), they were used in the present study.

### 1.2. The Influence of Domain-General Abilities and Domain-Specific Knowledge Depending on the Different Specific Mathematics Achievements

The description of the interaction between domain-general and domain-specific performance seems to be interesting. The strength of the influence varies depending on the sample’s age as well as the complexity of the mathematical demands (e.g., Fuchs et al. 2010; Lee and Bull 2016). Corresponding with the longitudinal study conducted by Geary et al. (2017), the relevance of past mathematical abilities for following mathematical competences increases with age. Numerical knowledge and arithmetic abilities are crucial for all ages, whereas fractional knowledge is defining for older grades. For younger grades, compared to older age groups, domain-general abilities are more important than domain-specific knowledge. On the other hand, domain-general abilities and domain-specific knowledge are equally relevant for older grades. With a particular focus on the visual working memory, Wang et al. (2022) demonstrate that the spatial working memory is involved in different ways depending on the degree of difficulty of open mathematical problems. Whereas the spatial working memory is rather associated with solving simple open mathematical problems, spatial visualization is linked to the solving of more difficult open mathematical problems. Earlier studies have already indicated the varying correlation of spatial working memory with simple and difficult mathematical tasks for closed(-ended) mathematical problems. Increasing difficulty of mathematical problems increases the demand of spatial visualization abilities (Manger and Eikeland 1998; Penner 2003). Similarly, depending on the specific mathematical branch that is being assessed, Liang et al. (2022 Jun) have found differing influences of the verbal and visual-spatial working memory on elementary school children’s mathematical abilities.

According to Raghubar et al. (2010), existing inconsistencies tend to be explained by features of the specific task and constructs of the working memory component. Assessing the link between different mathematical problems and the executive function, Best et al. (2011) interpret that solving problems depends on the students’ choice and application of a strategy as well as their self-monitoring. Calculating requires less executive control because, as Best et al. (2011) suggest, it rather relates to the retrieval of factual information. The result that the performance given in different developmental stages relates differently to executive function is addressed and assessed in several studies (Imbo and Vandierendonck 2007; McKenzie et al. 2003; Rasmussen and Bisanz 2005). It becomes evident that children’s age relativizes this connection. This permits the assumption that younger children access their working memory more strongly than older children when solving mathematical problems. Therefore, the sample’s age appears to play a significant role for the explanation of inconsistencies in the findings.

### 1.3. Predictive Links between Working Memory and Mathematical Processing

Before having a look at the links between working memory and mathematical processing, we will summarize results that highlight the connection between working memory and so-called elementary cognitive tasks (Carroll 1993). These tasks are referred to as “elementary” because they merely require basal cognitive processes instead of specific knowledge, such as mathematic knowledge, or prior experience. The assumption is that everyone should be able to solve elementary cognitive tasks successfully, provided they have enough time. Only a small number of mental processes have to be performed in order to reach the correct solution (Goecke et al. 2021). Nonetheless, as Goecke et al. (2021) list, different cognitive processes are involved: (sustained) attention, initial perception of stimuli, encoding, coding, updating and retrieval from working memory, reaction setup and execution of a motoric reaction (Ackerman and Kyllonen 1991; Kyllonen and Christal 1990). 

Regarding working memory, all theories assume that there is a limited capacity to the working memory. In other words, the possible amount of information that can be stored and processed by the working memory is limited (Baddeley 2012; Conway et al. 2008; Cowan 2005). This limitation is reflected in the working memory’s capacity, which is used to explain individual differences (Cowan 2010). Limits of this capacity are assumed to be the cause of low performance in cognitive tasks such as reasoning and decision-making. Wilhelm et al. (2013) show that people with a lower capacity are surpassed by those with a higher capacity in those tasks. It has been determined that working memory capacity is strongly linked to reasoning abilities (Kane et al. 2005; Kyllonen and Christal 1990; Oberauer et al. 2005) and is thus the core of reasoning abilities (Kyllonen and Christal 1990). As the complexity of elementary cognitive tasks increases, so do the demands on the working memory. Demands on working memory capacity in complex elementary cognitive tasks are presumed to be a causal factor which incrementally contributes to the relationship to cognitive ability. Mathematical demands may be understood as elementary cognitive tasks or complex elementary tasks that require mathematical knowledge. Accordingly, differences in processing mathematical demands need to be attributable to different working memory capacities.

A connection between working memory and processing of mathematical demands has long been assumed, but the evidence has yet to be supported by sufficient data. Automatized mathematical knowledge is advantageous in its quick availability and because it binds little to no cognitive resources. Thus, the automatization of mathematical knowledge is indicative of elaborated and processed knowledge. For only knowledge that is understood is sustainable and therefore retrievable in the long run. It becomes apparent that adults are more proficient at solving simple arithmetic tasks than children. Since children are not equipped with the same mathematical knowledge as adults, they require more time to solve the tasks and they employ less elaborated strategies such as counting, e.g., counting the smaller summand (e.g., Ashcraft 1992; Siegler 1988). As they age, children begin to use more efficient strategies to solve arithmetic problems. Imbo and Vandierendonck (2007) demonstrate that 10- to 12-year-old children need their working memory for strategies relating to retrieval, transformation and counting and that the ratio of available working memory resources and demands of the arithmetic tasks varied in the course of the children’s development. This change has also been demonstrated in various neuroscientific studies. Children activate more domain-general brain areas, which are associated with increased performance in attention and working memory. Adults, on the other hand, who can rely more on efficient solving strategies, such as the recall of numerical factual knowledge, show a focused neuronal activity of more specific areas for computation and memory (e.g., Kucian et al. 2008). A frequent use of retrieval, efficient memory recall and efficient counting processes reduces the demands on working memory. However, this also means that people, especially children, with less strong working memory functions have more difficulties acquiring and automatizing mathematical knowledge. For that purpose, Maehler and Schuchardt (2009) have assessed three groups with 27 children each, one group consisting of children with a normal IQ and learning disabilities (ICD-10: mixed disorders of scholastic skills), another of those with learning difficulties and a low IQ (intellectual disability) and a control group that is made up of children with an average development, normal performance in school and a normal IQ. Both groups with learning disabilities had an overall deficit in working memory. In a meta-analysis of 21 studies, Chen and Bailey (2021) investigated the consequences of conducting dual-task experiments. During these experiments, people have to solve two tasks at the same time, in this case a working memory task and a mathematical demand. If the working memory is involved with solving the arithmetic task, this will consequently lead to a reduction of working memory capacity. The results indicate that a higher load on the working memory results in a slower calculation of the arithmetic tasks. It is evident that the type of load on the working memory is the most substantial moderator. Load on the central executive slows down the performance the most.

### 1.4. Research Questions

The literature has shown that working memory influences mathematical competences (Friso-van den Bos et al. 2013; Gallit et al. 2018; Gathercole et al. 2005; Geary 2011; Lee and Bull 2016; Mazzocco and Kover 2007; Siegler et al. 2012; Toll et al. 2011), but that the type of prediction is age-dependent (Li and Geary 2013; Soltanlou et al. 2015; Van de Weijer-Bergsma et al. 2015). Moreover, previous findings indicate that the influence of domain-general skills depends on the specificity of mathematical performance itself (Ashcraft 1992; Chen and Bailey 2021; Imbo and Vandierendonck 2007; Siegler 1988). Additionally, there is evidence which highlights the relevance of cognitive performance for the resources available in different working memory components (Kane et al. 2005; Kyllonen and Christal 1990; Oberauer et al. 2005).

Based on these findings, the present study investigates the following research questions considering pre-school children’s intellectual abilities

1. Do the domain-general and domain-specific achievements differ according to the pre-school intellectual abilities?
2. Is there a difference in mathematical achievement between different intellectual ability groups in 2nd grade?
3. Which pre-school abilities predict mathematical achievement in 2nd grade within each group?
4. How do the different domain-general and domain-specific variables at pre-school predict mathematics at 2nd grade within each group?

## 2. Design, Materials and Methods

### 2.1. Design 

In order to gain in-depth insights into the relationship between intelligence, working memory and mathematical performance, groups of intellectual abilities are formed. These groups are examined in a longitudinal design throughout several times of measurement, starting in pre-school. This design aims to examine the development of children with low, average and high intellectual abilities. 

### 2.2. Participants

Within the German SCHUES-project (“Schulbezogene Umschriebene Entwicklungsstörungen (SCHUES)—Prävention und Therapie unter Einbezug neuronaler Korrelate und des Entwicklungsverlaufs”), a study funded by the Federal Ministry of Education and Research, 1868 children (908 girls and 960 boys) attending their second of three years of kindergarten were recruited. The study was approved by the Ethical Committee of University of Potsdam (approval number: 9/29). All parents gave written informed consent before data collection. At t1, children were tested in their second year at a local German kindergarten. The mean age of children was 63.0 months (*SD* = 4.4, range 49–81). On average nine months later (*SD* = 1.8, range 4–15), children were tested again in their final year of kindergarten with a mean age of 72.4 months (*SD* = 4.2, range 60–89). At the end of 2nd grade, which was attended by the children on average 24 months later (*SD* = 4.3, range 18–49), children were tested again. Some children stayed in kindergarten for an additional year or repeated 1st grade and therefore attended 2nd grade one year (*n* = 132) or two years (*n* = 5) later than the majority of the children (*n* = 1197). The mean age was 96.1 months (*SD* = 5.2, range 87–131). Most of the children (91.1%) spoke monolingual German at home. 

### 2.3. Procedure 

Children were tested individually in a quiet room at their kindergarten (t1 and t2) or their school (t3). The experimenters received comprehensive training delivered by senior project members. This included a two-day workshop, a videotaped testing session with a child as well as one supervised testing session at kindergarten or at school within the experimenter’s first week of testing. The videotaped trial testing and the supervised testing session were evaluated individually by a senior project member.

### 2.4. Tasks

#### 2.4.1. Numerical and Mathematical Skills (t1 and t2)

Numerical and mathematical precursor skills at t1 and t2 were assessed using a modified version of the neuropsychological test battery ZAREKI-K (Von Aster et al. 2009). The domain Counting maps the child’s ability to recite and apply the number word series. In terms of content, it is to be separated from the domain Number Knowledge, which summarizes the child’s ability to grasp a visual representation of numbers and to assign a quantity-related meaning to them. The domain Magnitude Estimation/Calculation describes the child’s ability to grasp quantities quickly and to compare them with one another. The task descriptions, the number of items and the maximum score of each subtest as well as results of confirmational factor analysis were described by Poltz et al. (2022). The T-values of the three math-related domains Counting, Number Knowledge and Magnitude Estimation/Calculation were added, standardized and transformed into T-values. We found a reliability coefficient of .88 for t1 and .80 for t2. 

#### 2.4.2. Mathematical Abilities (t3)

To assess children’s abilities to solve written addition and written subtraction problems, we used the subtests addition (Cronbach’s Alpha: .75) and subtraction (Cronbach’s Alpha: .68) of the standardized German mathematics test DEMAT 2+ (Krajewski et al. 2004). Both subtests contain written problems on a sheet of paper. Children received one point for a correct answer. We decided to sort item “?−19 = 15” to the scale addition because the problem is solved by using addition instead of subtraction. Therefore, the maximum scores were five points for addition and three points for subtraction. 

To assess children’s abilities to solve addition and subtraction problems in applied contexts, we used a selection of items of the subtest Calculation of the BUEGA (Esser et al. 2008). BUEGA is a German test battery to assess children’s performances relevant in school and to identify children with specific learning and developmental disorders in primary school. The subtest Calculation assesses a wide range of numerical and mathematical skills, e.g., counting, number knowledge and orally presented problems in applied contexts concerning basic mathematical operations. The material of the subtest calculation consists of a picture block. Every item is accompanied by a drawing. We selected items which assess applied contextual problems. Due to the mathematical curriculum of 2nd grade in Germany, we limited the selection to tasks that can be solved by addition and subtraction. The scale applied contextual problems consists of five items (e.g., “Julia counts her 50 Cents coins. How many coins does she need to buy an ice cream for 2 euros?”). 

Confirmatory factor analysis confirmed the three mathematical scales (addition, subtraction, and applied contextual problems). Results are displayed in Table 1. To evaluate the three-factor-solution, two alternative models were calculated: a two-factor-solution (factor 1: addition and subtraction, factor 2: applied contextual problems) and a one-factor-solution. Results of the alternative models as well as AIC and BIC are displayed in Table 1. The three-factor model showed the lowest values of AIC and BIC and thus favors this model. AIC and BIC take into account the model fit as well as the model economy (Geiser 2011). Cronbach’s alpha in this study is .73 for addition, .75 for subtraction (*n* = 1337) and .69 for applied contextual problems (*n* = 1350). 

#### 2.4.3. Visuospatial Sketchpad

A Corsi-Block task (Milner 1971) was used to assess children’s performance of the visuospatial sketchpad. Behavioral and neuropsychological evidence demonstrated correspondence between spatial short-term memory and performance in the Corsi-Block task (for a review, see Baddeley 2002). Previous studies demonstrated the applicability of the Corsi-Block task for children aged four or older (e.g., Bull et al. 2008; Passolunghi and Cornoldi 2008; Rasmussen and Bisanz 2005; Roebers and Zoelch 2005; Schmid et al. 2008). Six red wooden blocks were nailed on a wooden board (Roebers and Zoelch 2005; Schmid et al. 2008). The experimenter pointed to a sequence of two blocks. The child was instructed to point to the same two blocks in the same order. For each span, the experimenter showed two simple and two complex sequences before progressing to a span of three. The maximum span was five blocks. The child got one point for showing a correct sequence. The task was terminated after the child failed four consecutive trials. The maximum score was 16 points. Cronbach’s alpha in the present study is .83 (*n* = 1820) for t1 and .80 (*n* = 1652) for t2. The Corsi-Block task was found to correlate with Matrices (*r* = .50, *p* < .01) and hand movements (*r* = .71, *p* < .01), but not with the phonological loop (Schmid et al. 2008). Schmid et al. (2008) found a retest reliability (three weeks) of *r_tt_* = .61. 

#### 2.4.4. Phonological Loop

To assess children’s performance of the phonological loop, a shortened version of the subtest Phonological Working Memory for Artificial Words of the SETK 3-5 (Grimm 2001) was used. The recitation of artificial words is considered a pure method and is therefore often used to assess the phonological short-term memory (De Smedt et al. 2009; Schmid et al. 2008). Since unknown phonological sequences are used here, supporting representations of the long-term memory are reduced (cf., De Smedt et al. 2009). Children were asked to recall fantasy characters by repeating their names (artificial words) which the experimenter had read aloud once. Children received one point for correctly reproducing the artificial word. The maximum score is ten. Cronbach’s alpha in the present study was .70 (*n* = 1820) for t1 and .65 (*n* = 1651) for t2. The subtest was found to correlate *r* = .49 (*p* < .01) with Number Span and *r* = .65 (*p* < .01) with Word Span (Schmid et al. 2008).

#### 2.4.5. Central Executive

A dual-task (or complex span task) was used to assess children’s performance of the central executive (see, Schmid et al. 2008). Dual-tasks have a processing component that addresses executive control functions. Typically, auditory span or number span backwards are used. Auditory span tasks have already been used when examining children (e.g., Fuchs et al. 2010; Passolunghi 2011; Pickering and Gathercole 2001). Schmid et al. (2008) developed an auditory span task for pre-school children based on a simple perceptual judgment. For the present study, the task, originally developed for the use at a computer, was adapted into a paper-pencil version. Here, children were presented with two drawings of a two-syllable object for 1.5 s each. The objects were colored either blue or red. Children were asked to immediately point to a color card to indicate into which “color bucket” the object had fallen. After two objects, children were asked to repeat the names of the objects. After four items with two objects, four items consisting of three objects each and four items consisting of four items each were presented. The task was terminated when three wrong answers were given within a span. Children received one point for pointing to the correct color card and recalling the objects’ names correctly. This procedure deviates from Schmid et al. (2008). It had become apparent that some children refrained from the perceptual judgment or always randomly pointed to the color cards. These children only repeated the object names immediately after the end of the sequence. Since no central executive, but only phonological processes, were involved in these cases, only items that indicated both a perceptual judgment as well as a memory performance were judged to be correct. The order in which the object names were recalled was not relevant. The maximum score was 12. Cronbach’s alpha was .72 (*n* = 1809) and .72 (*n* = 1652). Retest reliability with an interval of three weeks was *r_tt_* = .80 (*p* < .01; Schmid et al. 2008). Auditory span did not correlate with color span backwards. In addition, factor analysis did not reveal a third factor. Auditory span loaded equally high on the phonological tasks and tasks addressing visuo-spatial working memory (Schmid et al. 2008). 

We ran a confirmatory factor analysis to confirm a three-factor-structure of the three working memory tasks. The model of t1 as well as t2 fitted the data well. Results are displayed in Table 2. To evaluate the three-factor structure, an alternative model was calculated. A one-factor solution seemed plausible. Results of the alternative model at t1 and t2 as well AIC and BIC are displayed in Table 2. The three-factor models showed the lowest values of AIC and BIC and thus favor these models.

#### 2.4.6. General Intelligence

The subtests Nonverbal Intelligence and Verbal Intelligence of the BUEVA-III (Esser and Wyschkon 2016) were used to assess children’s general intelligence. The BUEVA-III is a German test battery to assess children’s stages of development and to identify children at risk for later developmental disorders before school entry. 

The subtest Nonverbal Intelligence assesses children’s ability of reasoning by analogy and logical thinking. Children have to identify the odd picture out of a set of pictures. With one point for each correct answer, the maximum score is 25 for four-year-olds and 28 for children aged five years and older. Cronbach’s Alpha is .87 (Esser and Wyschkon 2016). Children’s performance in the subtest Nonverbal Intelligence has been shown to correlate *r* = .54 (*p* < .001) with their performance in the Coloured Progressive Matrices (Bulheller and Häcker 2002).

The subtest Verbal Intelligence assesses children’s ability of verbal reasoning by creating analogies. Children are asked to complete orally presented sentences. With one point for each correct answer, the maximum score is 19 for four-year-olds and 23 for children aged five years and older. Cronbach’s Alpha is .87 (Esser and Wyschkon 2016). The subtest Verbal Intelligence has been found to correlate *r* = .71 (*p* ≤ .001) with the subtest Identify Terms of the HAWIVA-III (Ricken et al. 2007). 

To identify three intellectual ability groups, we used one standard deviation as cut-off-criteria. Children were assigned to one ability group if they met the criteria on both occasions. We identified children as having low intellectual ability if they scored below a T-value 40 in general intelligence at t1 and t2. We identified children as having average intellectual ability if they showed T-values between 40 and 59 at t1 and t2, and children as having high intellectual ability if they showed a T-value of 60 or higher (see Esser et al. 2008 for interpretation guidelines). Hence, analyses regarding intellectual ability groups are based on 1209 children (614 boys, 595 girls). A number of 166 children had to be excluded as there was only one value of intellectual ability. Another 493 children were excluded because they did not fall into the same intellectual ability group at both time points. Drop-out analysis with all variables showed a very small, but significant difference (Bonferoni corrected at *p* < .002) in children’s general intelligence at t2 (*t*(841.93) = 5.97, *p* < .001, *r* = .04), indicating a higher intelligence of children excluded (*M* = 53.0, *SD* = 10.33) from further analyses than children included (*M* = 49.78, *SD* = 9.34). No other differences could be found.

#### 2.4.7. Visual Attention

The subtest Attention of the BUEVA-III (Esser and Wyschkon 2016) was used to assess children’s ability of visual attention. The subtest Attention aims to evaluate children’s ability to maintain visual attention by assessing the speed and accuracy with which they can identify two target pictures from a range of different pictures. Children were asked to go through the pictures, line by line, and mark every picture that depicted the target images (dog or elephant). After 90 s, the test was terminated. A score for Visual Attention was computed by subtracting the total number of wrong answers from the number of correct answers. Split-half reliability is .88 (Esser and Wyschkon 2016). 

### 2.5. Data Analysis

Correlational analyses were conducted using SPSS version 28. Path analysis with manifest variables was used to analyze our theoretical models. This was done using the full maximum likelihood method (FIML) in MPLUS 7.1 (Muthèn and Muthèn 2013). FIML estimates missing values directly without imputing them for each individual parameter (Enders 2001). A basic model was analyzed and all relevant parameters were estimated. Non-significant paths were removed step by step. 

## 3. Results

Descriptive statistics of each tested variable for the total sample are presented in Table 3. All skewness scores were within an acceptable range (Miles and Shevlin 2001). However, the distribution of addition at t3 shows a floor effect. 

### 3.1. Correlational Analyses

Due to the large number of variables in this study, we split the intercorrelations into two tables. Firstly, Table 4 presents intercorrelations between the variables at t1 and t2 only. Intercorrelations of variables at t1 are displayed below the diagonal and the intercorrelations between variables at t2 above the diagonal. Results showed a strong relationship between intelligence and mathematical/numerical precursor skills at both time points before school. Furthermore, there were moderate relationships between intelligence and the three working memory components as well as attention. The weakest relationship was found between the two domain-general skills, phonological loop and attention. 

Secondly, Table 5 presents correlations between variables across the three time points. Results indicated a high stability of intelligence and mathematical/numerical precursor skills from t1 to t2. Domain-general skills demonstrated a medium to high stability before school. 

What was already evident cross-sectionally could also be found across time points: There was a strong relationship between intelligence and mathematical/numerical precursor skills across t1 and t2. 

Furthermore, we found a moderate relationship between mathematical/numerical precursor skills at pre-school (t1 and t2) and the three mathematical tasks at t3. Among the pre-school domain-general skills, intelligence (t1 and t2) showed the highest correlations with mathematical/numerical precursor skills at t1 and t2 as well as with the mathematical tasks at t3. 

Interestingly, relationships between pre-school variables and applied contextual problems were consistently higher than relationships between pre-school variables and addition and subtraction. Thus, pre-school domain-general and domain-specific skills seem to be of different significance depending on the type of mathematical tasks solved at school.

### 3.2. Comparison of Performance between Intellectual Ability Groups

To answer the question if children’s (1.), pre-school domain-general and domain-specific achievements and (2.), mathematical achievement in 2nd grade differ according to their pre-school intellectual abilities, a one-way independent ANOVA was conducted. Results showed significant differences in all variables at t1, t2 and t3 between the three intellectual ability groups (see Table 6). Cohen’s (1988) conventions were used to evaluate the effect sizes (.01 = low effect, .06 = medium effect, .14 = large effect).

**Figure 1:**
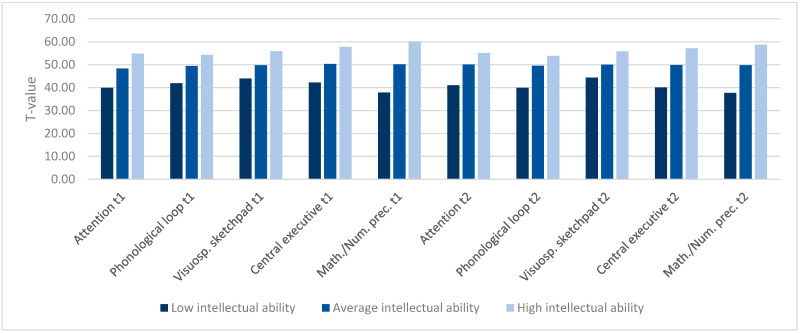
Comparison of achievements in variables t1 and t2 between low, average and high intellectual achievement groups. *p* ≤ 0.001 for all comparisons between groups.

(1) There was a high main effect of group on mathematical/numerical precursor skills (domain-specific) at t1 and t2 (see Figure 1). Post-hoc tests (Hochberg) revealed significant differences (*p* < 0.001) between the three intellectual ability groups at t1 and t2. Children with low intellectual ability showed lower scores on mathematical/numerical precursor skills than children of average intellectual ability, who in turn scored lower than children of the high intellectual ability group. Furthermore, we found medium to high effect sizes for all domain-general skills at t1 and t2. Post-hoc tests (Hochberg and Games-Howell respectively) revealed the lowest achievement scores in all domain-general skills for children of the low intellectual ability group, followed by children of the average intellectual ability group. The highest scores in all domain-general skills were achieved by children of the high intellectual ability group.

We found an overly high drop-out in the low intellectual ability group at t3. More than half of the children with low intellectual ability could not be tested again at t3. Drop-out analyses with variables at t1 in this group only revealed significant lower general intelligence of children who could not be tested (*M* = 31.88, *SD* = 4.60, *n* = 80) than children who could be tested at t3 (*M* = 33.44, *SD* = 4.67, *n* = 68; *t*(146) = -2.05, *p* = .042, *r* = .17).

**Figure 2:**
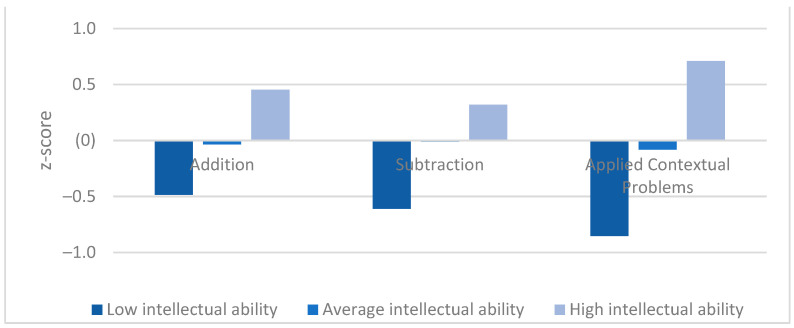
Comparison of achievements in mathematical tasks at t3 between low, average and high intellectual achievement groups. *p* ≤ 0.01 for all comparisons between groups.

(2) There were low (addition and subtraction) to medium effects (applied contextual problems) on the mathematical achievements in 2nd grade (see Figure 2). Post-hoc tests (Games-Howell) revealed that children of the low intellectual ability group scored lower on three mathematical tasks than children of the average intellectual ability group, who in turn scored lower than children of the high intellectual ability group. The differences between the three intellectual ability groups in addition (*p* < .001), subtraction (*p* < .01) and applied contextual problems (*p* < .001) were significant.
(3) A look at Figure 2 revealed that the three mathematical tasks seem to be differentially demanding for the three intellectual ability groups. Children of the low intellectual ability group showed the lowest achievement in applied contextual problems. This performance was significantly lower than that in written addition (*t*(67) = 6.159, *p* < .001) and in written subtraction (*t*(67) = 2.588, *p* = .012). No difference was found between children’s performance in written subtraction and addition (*t*(67) = 1.190, *p* = .238). In contrast, children of the high intellectual ability group showed the highest performance in applied contextual problems. This performance was significantly higher than that in written subtraction (*t*(122) = −3.547, *p* < .001) or written addition (*t*(122) = −2.534, *p* = .013). Again, no difference was found between written subtraction and addition (*t*(122) = 1.280, *p* = .203). Regarding the performance of children in the average intellectual ability group, the lowest performance was found in applied contextual problems. This performance was marginally significantly lower than their performance in written subtraction (*t*(707) = 7.873, *p* = .062) and slightly but not significantly lower than their performance in written addition (*t*(707) = 1.386, *p* = .166). Again, we could not find differences in the achievement between written addition and subtraction (*t*(707) = −0.609, *p* = .542).

### 3.3. Path Analyses

To answer the question (3.), which pre-school abilities predict mathematical achievement in 2nd grade, and question (4.), how the different domain-general and domain-specific variables at pre-school predict mathematics in 2nd grade, path analyses were calculated for each intellectual ability group separately.

#### 3.3.1. Low Intellectual Ability Group

**Figure 3:**
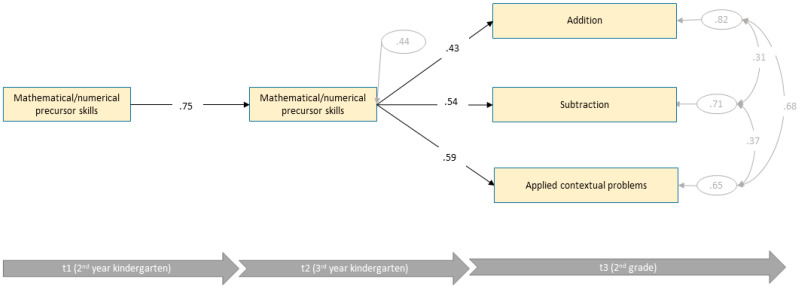
Final path model of the low intellectual ability group with standardized path coefficients (*n* = 131).

Figure 3 shows the estimates of the final path model. The model showed an acceptable data fit (*χ*^2^ (3) = 6.02, *p* = .111, CFI = .99, RMSEA = 0.088, SRMR = .041). Nevertheless, all domain-general skills had to be removed from the final model due to non-significant paths. Hence, for children of the low intellectual ability group, only domain-specific skills were able to predict later mathematical/numerical skills and explained 56% of variances. Only mathematical/numerical precursor skills accounted for variances of three mathematical scales in 2nd grade. Applied contextual problems were most dependent of mathematical/numerical precursor skills (*R*^2^ = 35%), followed by subtraction (*R*^2^ = 29%) and addition (*R*^2^ = 18%). Compared to the models of the average and high intellectual ability groups, mathematical/numerical precursor skills accounted for the highest explanation of variance. 

#### 3.3.2. Average Intellectual Ability Group

**Figure 4:**
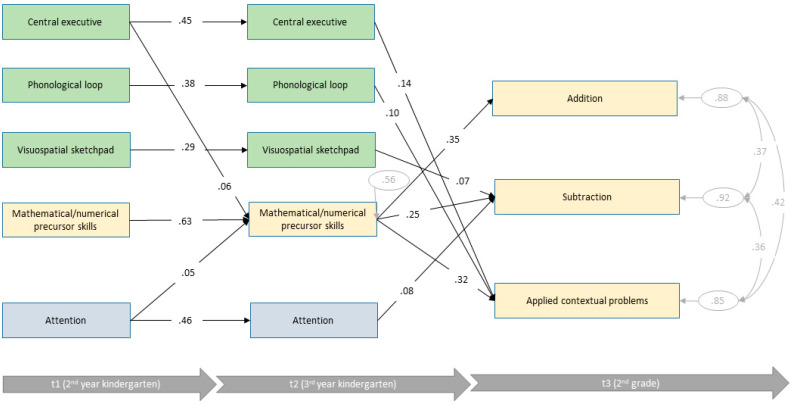
Final path model of average intellectual ability group with standardized path coefficients (*n* = 908).

Figure 4 shows the estimates of the final path model of the average intellectual ability group. This more complex model fitted the data well (*χ*^2^ (51) = 267.52, *p* < .001, CFI = .89, RMSEA = .068, SRMR = .072). Mathematical/numerical precursor skills at t2 of children with average intellectual ability could be explained mainly by mathematical/numerical precursor skills one year before. In addition, central executive (*β* = .06) and attention (*β* = .56) could explain a very small but significant amount of variance of mathematical/numerical precursor skills at t2. Overall, a total of *R*^2^ = 44% could be explained. Regarding 2nd grade, mathematical/numerical precursor skills were consistently the best predictors of later mathematical achievements at t3. Interestingly, the involvement of domain-general skills varied among the different mathematical tasks. Achievement in addition did not depend on previous domain-general skills (*R*^2^ = 12%). Visuospatial sketchpad and attention were additional predictors of subtraction. But, taken together, domain-general and domain-specific skills could explain only a very small amount of variances (*R*^2^ = 8%). In contrast, applied contextual problems were predicted by phonological loop and central executive in addition to domain-specific skills (*R*^2^ = 15%). 

#### 3.3.3. High Intellectual Ability Group

**Figure 5:**
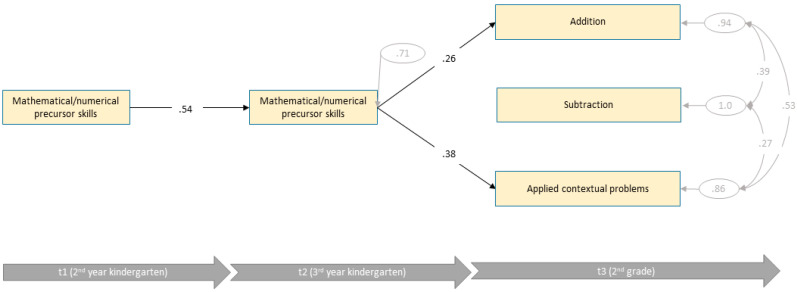
Final path model of high intellectual ability group with standardized path coefficients (*n* = 135).

Figure 5 shows the estimates of the final path model of the high intellectual ability group. Again, the model showed an acceptable data fit (*χ*^2^ (4) = 6.43, *p* = .169, CFI = .98, RMSEA = .067, SRMR = .066). Similar to the estimated model of the low intellectual ability group, all domain-general skills had to be removed from the final model due to non-significant paths. Hence, only domain-specific skills were able to predict later mathematical/numerical skills (*R*^2^ = 29%). However, the explained variance was much lower than for children with low intellectual ability. Only addition (*R*^2^ = 6%) and applied contextual problems (*R*^2^ = 24%) could be predicted by previous mathematical/numerical precursor skills. Neither domain-general nor domain-specific skills were capable of predicting subtraction. 

#### 3.3.4. Summary of Findings

Overall, domain-specific skills influenced later mathematical skills, independent of children’s intellectual ability group. However, only children with average intellectual ability additionally relied on cognitive resources of domain-general skills. Here, the relationships between different domain-general abilities and later mathematical achievement were affected by the type of mathematical task. 

## 4. Discussion

This study investigated the influence of pre-school domain-general predictors (i.e., different components of working memory and attention) on domain-specific performances (i.e., mathematical achievement) while controlling for cognitive performances by examining three different intellectual ability groups.

### 4.1. Children’s Domain-General and Domain-Specific Achievement Differs according to Their Intellectual Ability Group

The results showed that children with diverse intellectual abilities (low—average—high) already started with significant differences in their domain-general achievement one year before entering school. Thus, different predictive strengths of influence already existed before school entry. Children with low intellectual abilities showed the lowest achievements in all domain-general predictors as well as domain-specific numerical and mathematical tasks. Children of average intellectual ability performed significantly better in all areas compared to their peers with lower intellectual ability. Children of high intellectual abilities demonstrated significantly higher performance than the average group in both domain-general and domain-specific tasks. The literature revealed that the capacity of working memory and cognitive abilities are closely related (Kyllonen and Christal 1990). Furthermore, previous research suggested that limitations of working memory capacity led to limitations of achievements in cognitive tasks (Kane et al. 2005; Kyllonen and Christal 1990; Oberauer et al. 2005). The results of our study extend these findings. Accordingly, children with different intellectual abilities showed significantly different performance of working memory. Moreover, in line with the literature (Friso-van den Bos et al. 2013; Gallit et al. 2018; Gathercole et al. 2005; Geary 2011; Lee and Bull 2016; Mazzocco and Kover 2007; Siegler et al. 2012; Toll et al. 2011), we already found significantly different mathematical performances one year before school entry. Thus, children’s intellectual ability group was closely related to their level of working memory and mathematical performance, e.g., children with low intellectual ability were particularly weak in domain-general and domain-specific abilities.

Results of previous longitudinal studies support the assumption of differing mathematical achievement in 2nd grade between children with different intellectual abilities assessed in pre-school (Friso-van den Bos et al. 2013; Gallit et al. 2018; Gathercole et al. 2005; Geary 2011; Lee and Bull 2016; Mazzocco and Kover 2007; Siegler et al. 2012; Toll et al. 2011). We found significant differences regarding children’s mathematical skills approximately 3 years after the first assessment. Children with low intellectual abilities demonstrated the weakest mathematical performance in 2nd grade. They were followed by children with average intellectual abilities, who scored significantly better in the administered mathematical tasks. The significantly highest mathematical achievement was reached by children with high intellectual abilities. This highlights, firstly, that we were already able to assign children to differing intellectual ability groups in pre-school. Children with differing intellectual abilities showed different domain-general skills before school entry and therefore did have different prerequisites for acquiring school-relevant skills. Secondly, differences in domain-specific skills, here mathematical skills, still existed after three years. This raises the question if children with differing intellectual abilities rely on different domain-general skills to solve mathematical problems.

### 4.2. The Relevance of Domain-General Abilities to Predict School Mathematics Changes according to Children’s Intellectual Ability

Results of path analyses revealed that the relevance of pre-school predictors changes depending on children’s intellectual ability. The path models of the intellectually weakest and strongest groups showed rather simple predictive structures. In contrast, a more complex predictive model was found for the average intellectual ability group. Comparing the explained variances between the three ability groups in 2nd grade, the model of the low intellectual ability group could explain up to 35% of the variance by only one predictor: numerical and mathematical skills in pre-school. Domain-general and domain-specific pre-school variables explained a much smaller amount of up to 15% of the variance of mathematical domains for the average intellectual ability group in 2nd grade. Nonetheless, all mathematical domains were predicted here as well. Furthermore, up to 14% of the variance was explained for children with high intellectual ability, but only for two out of three mathematical domains.

The third research question focused primarily on domain-general and domain-specific predictors for children with different intellectual abilities. Results showed that different intellectual ability groups were found to demonstrate a different predictive pattern. The most complex model held for the average intellectual ability group, with all pre-school variables playing a significant role in predicting 2nd grade math achievement. The simplest models were established by the low and high intellectual ability groups. Although the exclusive predictive effect derived from pre-school numerical skills in both models, the origin of this predictive association needs to be interpreted differently. Comparisons of children’s domain-general and domain-specific achievements showed that the two groups were fundamentally different. Already at t1, children with low intellectual abilities showed a significantly weaker performance of up to 1.5 standard deviations in domain-general predictors compared to children with high intellectual abilities. However, the literature revealed that younger children mainly use working memory to solve mathematical problems (Friso-van den Bos et al. 2013). Since both groups of children were of the same age and each worked on identical mathematical tasks, this raises the question of why both groups showed relatively similar models in spite of their different initial levels of domain-general resources. We assume that children’s weak pre-school domain-general resources at t1 could already explain why there were no predictive effects of any working memory task, nor of attention. As a result, these very weak domain-general capacities could not be used to solve mathematical tasks. Lee and Bull (2016) emphasized that early numerical skills are an important predictor as well. Children with insufficient domain-general resources only have the possibility to rely on already acquired numerical and mathematical competencies in order to cope with mathematical problems later on in their development. Likewise, Lee and Bull (2016) described that higher working memory capacities in pre-school predict higher rates of growth in mathematics. The present study did not examine the effect on the growth rate of mathematics achievement. However, comparing the mathematical competencies in 2nd grade revealed that the achieved mathematical performances differed between the low and high intellectual ability group by about 1 to 1.5 standard deviations. Thus, very different mathematical performance levels were reached.

Children with high intellectual abilities also demonstrated a very simple predictive model, although these children had been able to rely on very good domain-general resources throughout their development, i.e., for at least one year before school entry. As described previously by Lee and Bull (2016), there is a stable effect of working memory across grades on mathematical performance. As a result, the relevance of past mathematical performance increases over the years. Following this line of argumentation, children with higher capacity of working memory would be able to acquire more mathematical skills than children with lower domain-general resources. This means that, although children with high intellectual abilities naturally had these resources available in 2nd grade, this group no longer had to rely on them. It seems that the mathematical competencies already acquired are sufficient to enable this group to cope with the mathematical tasks in 2nd grade. However, this assumption needs to be verified in further studies starting much earlier in children’s development.

A complex predictive model was found for children with average intellectual abilities. Based on the literature, we expected this complexity regarding the influence of domain-general predictors on mathematical achievement. All components of working memory as well as attention were found to affect later mathematical performance in 2nd grade. However, even here, the influence of earlier numerical and mathematical skills on later mathematical achievement appeared to be rather high. This became very obvious between the first two measurement points in pre-school. The same was true for pre-school to 2nd grade. Thus, the present study supports the assumption that domain-general predictors, here especially working memory, play a rather important role in the mastery of mathematical tasks. However, previously acquired mathematical skills and thus domain-specific predictors are even more relevant. This is in line with previous longitudinal studies highlighting the importance of early numerical skills for the development of arithmetical skills in school (e.g., Aunio and Niemivirta 2010; Fritz et al. 2013, 2018; Gallit et al. 2018; Poltz et al. 2022; Toll et al. 2016).

The literature indicated that younger children rely more strongly on working memory resources than older children when solving mathematical problems (Friso-van den Bos et al. 2013). According to the present study, this statement held true for children with average intellectual ability. These children were performing mathematical demands of 2nd grade by using their average domain-general skills in addition to their mathematical knowledge. The assumption did not apply to intellectually low-performing children who did not have sufficient working memory and attention resources. Likewise, the assumption did not apply to intellectually high-achieving children who demonstrated very good domain-general resources but who had additionally acquired very good mathematical knowledge early on that could be built upon sufficiently. Hence, it appears that, in addition to children’s age, it is primarily children’s cognitive performance that affects the structure of predictive models.

There were interrelations between children’s intellectual abilities on the one hand and working memory and attention on the other. Accordingly, predictive models of mathematical skills could not be interpreted without considering children’s levels of intellectual ability. Therefore, future studies should always consider children’s intellectual abilities, especially of those performing below and above average. This would only be possible with larger samples. If the sample size does not allow a distinction between intellectual levels, then the interpretations of such models will primarily apply to children with average intellectual abilities. Interpretations for all children, including the cognitive extreme groups, should not be drawn from this. 

### 4.3. Different Mathematical Tasks Require Different Domain-General Resources

The last research question brought the 2nd graders’ mathematical achievements and the respective predictors into focus. The underlying assumption was that increasing complexity of the tasks would increase working memory demands (see Goecke et al. 2021). Accordingly, not only (complex) elementary cognitive tasks, but also more demanding mathematical tasks should be predicted more strongly by the working memory than less demanding mathematical tasks.

Results of the confirmatory factor analysis confirmed that addition, subtraction and applied contextual problems represented three differing mathematical tasks. Furthermore, results indicated that the mathematical tasks were differentially demanding for children with different intellectual abilities. For children with high intellectual abilities, solving applied contextual problems seemed to be less demanding than solving addition and subtraction tasks. In contrast, for children with low intellectual abilities, applied contextual problems seemed to be more demanding than addition and subtraction. A similar but only marginally significant effect was found for the average intellectual ability group. Path analyses revealed that for children with average intellectual ability, pre-school numerical and mathematical skills were apparently not sufficient to solve applied contextual problems, which seemed to be more demanding for this group. Solving applied contextual problems was supported by the central executive as well as the phonological loop. In contrast, solving subtraction and addition tasks seemed to be less demanding for children with average intellectual abilities. Support from the central executive and phonological loop was no longer required. Only the working memory subsystem of the visuospatial sketchpad was needed for subtraction tasks. Accordingly, our findings for children with average intellectual abilities support prior findings that more demanding mathematical tasks require more working memory resources and less demanding tasks require less working memory resources (see Goecke et al. 2021). However, whether mathematical tasks were demanding for children depended on children’s mathematical abilities. This means that children with high mathematical abilities need other tasks adapted to their mathematical abilities to be demanding than children with low mathematical abilities.

A look at mathematical achievements between intellectual ability groups revealed that children with high intellectual ability scored up to one standard deviation above children with low intellectual ability. We can assume that for children with high intellectual ability, the three types of mathematical tasks used in this study were not very demanding and therefore did not require support from working memory. Consequently, pre-school numerical and mathematical skills adopted the predicting role and prepared children for solving these tasks. For children with low intellectual abilities, applied contextual problems seemed to be the most demanding mathematical tasks. However, compared to children with average and high intellectual abilities, these children seemed to struggle with addition and subtraction too. Unlike children with average intellectual abilities, no support from their working memory nor attention was evident.

Accordingly, the load caused by the mathematical task was not the same for every child of a specific age. Depending on their intellectual abilities, children developed different mathematical proficiency, with children of the high intellectual ability group displaying the highest mathematical achievement. In other words, children with high intellectual ability exhibited the highest degree of mathematical proficiency. As a result, depending on the degree of children’s mathematical proficiency, mathematical tasks would be of varying demands (Fritz et al. 2013). This, in turn, influenced the model of predictors. This is of high interest for future research. Intellectual abilities determine domain-general resources (such as working memory and attention) as well as domain-specific resources and as a result the developmental trajectories of mathematical skills. Along with the degree of mathematical proficiency, the difficulties of these tasks experienced by groups with different mathematical abilities and the resulting load vary. Children with high mathematical skills display a lower load when working on identical tasks compared to children with rather average mathematical skills. This, in turn, influences the relevance of pre-school domain-general skills to predict later mathematical skills. Looking at the results of the present study, children with higher mathematical skills drew on their working memory less than children with average mathematical skills when solving identical tasks.

### 4.4. Limitations

General intelligence showed a high stability rate in pre-school. Nevertheless, we decided on using children’s results of intelligence testing of both time points in pre-school to control for testing effects. Thus, 35% of participants could not be assigned to a particular intellectual ability group and were not included in further analyses. However, drop-out analyses revealed only a very low difference in children’s general intelligence at t2, indicating a higher intelligence of excluded children. However, there is no reason to assume that the conducted analyses were affected by this small effect. 

Furthermore, we found a considerable drop-out-rate in the low intellectual ability group over the three-year course. Drop-out-analyses of variables at t1 revealed general intelligence as a differing variable. Children who dropped out were found to have lower general intelligence. The reason may be that especially children with very low intelligence were not able to reach 2nd grade during the time the study was running, that means even after a one-year delay. Thus, analyses of children’s achievement in 2nd grade lacked children with very low intellectual abilities. It had to be assumed that the low to medium effects between the three intellectual ability groups would have been much higher if we had been able to consider children who dropped out as well. 

Using one standard deviation to identify three sampling groups causes a reduced variance in both extreme intellectual ability groups. Nonetheless, these samples are still sufficiently large and stable enough to allow corresponding models to be calculated. In addition, this could be an interesting approach for further research. We assume that a comparison of the models with mathematical demands of varying difficulty (e.g., tasks that are too easy, appropriate, and too difficult for high-achieving children) should lead to comparable results found for children with low, average, and high intellectual ability in this study. This would open up a possibility to prove if the influence of the predictors was primarily determined by content-related mathematical demands and less by statistical variance.

The achievement in the three working memory domains was measured by using one task for each domain. Because of the comprehensive study design, a much broader capture of working memory performance was not possible. The selection of the respective tasks was based on intensive research of available tests and examination of their reliability and overall goodness criteria with regard to the planned age group. Nonetheless, this might have led to a low reliability and impurity of each working memory domain. In addition, Miyake et al. (2000) already emphasized the importance of looking at both unity and diversity of cognitive functions. Their analyses suggested that latent variables might be a useful approach to examine the relevance of cognitive functions. This implies for future studies to have a special focus on the role of cognitive performance. In addition, however, these cognitive performances should be measured extensively enough to be able to form latent variables and use them in models.

To identify three intellectual ability groups, we used one standard deviation as the cut-off criteria. This resulted in a bigger sample of children with average intellectual abilities than children with low or high intellectual abilities. The more complex model of the average ability group found in this study could be the result of these sampling size differences. Therefore, we randomly reduced the sample of the average intellectual ability group by approximately 80%. We reran the analysis and could replicate the finding that children with average intellectual ability not only rely on previous numerical and mathematical abilities but on domain-general abilities as well.

Lastly, we found a small effect of age between the three intellectual ability groups. Children of low intellectual ability were found to be the oldest. This may be due to the age-groups used for the standardized tests for assigning T-values to children’s raw scores.

## 5. Conclusions

Depending on their intellectual abilities, children showed different domain-specific and domain-general abilities in pre-school as well as different mathematical achievement in 2nd grade. Interestingly, the three mathematical tasks were differentially demanding depending on children’s intellectual ability. 

Our findings should have consequences for teaching mathematics at school. In order to challenge children with high intellectual abilities, those children need mathematical tasks suitable to their abilities. We assume that only then do children need to draw on their cognitive resources, which enables them to train them. The same is true for children with low intellectual abilities and hence low working memory resources. If the tasks were too difficult, it is assumed that those children would not be able to access their resources. It would have to be examined in follow-up studies how children with low performance draw on their working memory resources when confronted with mathematical demands that require mathematical competences at their average level of abilities. We expect these children to use their working memory resources again once the tasks that they are presented with make suitable mathematical demands. This would, in turn, have the effect that the children would train their domain-general resources by using them. However, this link is merely a speculation and would thus need to be verified.

Furthermore, in studies where the developmental trajectories of mathematical skills are examined, it is important to make detailed observations and especially consider children’s intellectual abilities. Intellectual abilities determine how children perform in working memory, which in turn determines how children develop mathematical skills. Whether a mathematical task is demanding for an individual child depends on the developmental level of mathematical competence.

## Figures and Tables

**Table 1:** Results of confirmatory factor analyses of the three mathematical scales at t3 (*n* = 1350).

Model	*χ*^2^ (*df*), *p*	RMSEA (90% CI: Lower/Upper)	CFI	AIC	BIC
3-factor	214.45 (62), <0.001	0.04 (0.04/0.05)	0.96	16,617	16,836
2-factor	663.76 (64), <0.001	0.08 (0.08/0.09)	0.85	17,062	17,270
1-factor	826.94 (65), <0.001	0.09 (0.09/0.10)	0.81	17,223	17,426

AIC = Akaike’s Information Criterion. BIC = Bayesian Information Criterion.

**Table 2:** Results of confirmatory factor analyses of the three working memory tasks at t1 (*n* = 1825) and t2 (*n* = 1655).

	Model	*χ*^2^ (*df*), *p*	RMSEA (90% CI: Lower/Upper)	CFI	AIC	BIC
t1	3-factor	2003.73 (662), <0.001	0.03 (0.03/0.03)	0.89	48,635	49,280
	1-factor	4983.99 (665), <0.001	0.06 (0.06/0.06)	0.60	51,609	52,238
t2	3-factor	1288.93 (662); <0.001	0.02 (0.02/0.03)	0.92	50,313	50,946
	1-factor	3717.60 (665), <0.001	0.05 (0.05/0.05)	0.61	52,736	53,353

AIC = Akaike’s Information Criterion. BIC = Bayesian Information Criterion.

**Table 3:** Descriptive Statistics.

	*n*	*M*	*SD*	Range	Skewness
Total					
**t1**					
Age (months)	1868	62.98	4.35	49–81	0.42
Intelligence	1866	49.15	10.08	19–69	−0.07
Attention	1856	48.19	9.13	20–60	−0.56
Phonological Loop	1823	49.19	8.57	23–60	−0.54
Visuospatial sketchpad	1821	49.75	9.88	21–81	0.05
Central executive	1809	50.05	9.74	28–81	0.17
Mathem./numerical precursor skills	1808	49.91	10.01	17–84	0.01
**t2**					
Age (months)	1705	72.36	4.20	60–89	0.41
Intelligence	1704	50.70	9.74	15–69	−0.25
Attention	1698	49.73	8.63	20–60	−0.67
Phonological Loop	1651	49.15	8.71	19–60	−0.69
Visuospatial sketchpad	1655	50.38	9.86	19–78	−0.07
Central executive	1652	49.94	9.70	24–74	−0.01
Mathem./numerical precursor skills	1647	49.87	10.03	18–80	−0.05
**t3**					
Age (months)	1348	94.79	4.08	79–111	0.35
Addition	1337	0.00	1.00	−0.81–2.75	1.17
Subtraction	1337	0.00	1.00	−1.26–1.21	−0.07
Applied contextual problems	1350	0.00	1.00	−1.22–2.11	0.38

Variables of t1 and t2 were reported in T-values. Variables of t3 were reported in z-scores.

**Table 4:** Intercorrelations (Pearson) between variables t1 (below diagonal) and between variables t2 (above diagonal) (*n*).

		1	2	3	4	5	6
1	IQ	-	0.40 (1696)	0.39 (1649)	0.24 (1653)	0.42 (1650)	0.50 (1645)
2	Attention	0.46 (1855)	-	0.21 (1648)	0.30 (1652)	0.29 (1650)	0.37 (1646)
3	Phonological loop	0.40 (1822)	0.21 (1814)	-	0.22 (1651)	0.40 (1648)	0.39 (1642)
4	Visuospatial sketchpad	0.31 (1821)	0.31 (1814)	0.24 (1819)	-	0.28 (1652)	0.36 (1646)
5	Central executive	0.41 (1809)	0.29 (1803)	0.35 (1807)	0.31 (1808)	-	0.43 (1644)
6	Math./Numeric. precursor skills	0.57 (1807)	0.42 (1802)	0.40 (1806)	0.43 (1807)	0.43 (1800)	-

All correlations were significant (*p* ≤ 0.001). Numbers of cases are in parentheses.

**Table 5:** Pearson Correlations across the three time points: t1, t2 and t3 (*n*).

			t2					t3			
			1	2	3	4	5	6	7	8	9
**t1**	1	IQ	0.71 (1702)	0.40 (1696)	0.39 (1649)	0.24 (1653)	0.42 (1650)	0.50 (1645)	0.20 (1337)	0.23 (1337)	0.42 (1350)
	2	Attention	0.46 (1855)	0.52 (1690)	0.22 (1644)	0.29 (1647)	0.28 (1644)	0.36 (1641)	0.14 (1334)	0.19 (1334)	0.23 (1347)
	3	Phonological loop	0.40 (1822)	0.18 (1666)	0.48 (1624)	0.13 (1627)	0.34 (1624)	0.31 (1619)	0.08 (1304)	0.08 (1304)	0.23 (1317)
	4	Visuospatial sketchpad	0.31 (1821)	0.30 (1664)	0.24 (1819)	0.34 (1624)	0.29 (1621)	0.35 (1618)	0.20 (1305)	0.13 (1305)	0.24 (1318)
	5	Central executive	0.41 (1809)	0.24 (1657)	0.17 (1621)	0.22 (1616)	0.53 (1614)	0.36 (1611)	0.14 (1297)	0.16 (1297)	0.26 (1310)
	6	Math./Numeric. precursor skills	0.57 (1807)	0.38 (1657)	0.34 (1613)	0.38 (1616)	0.46 (1614)	0.74 (1611)	0.35 (1297)	0.32 (1297)	0.48 (1310)
**t3**	7	Addition	0.24 (1274)	0.15 (1271)	0.15 (1239)	0.15 (1241)	0.21 (1240)	0.37 (1237)	-	0.45 (1337)	0.52 (1337)
	8	Subtraction	0.24 (1274)	0.20 (1271)	0.15 (1239)	0.20 (1241)	0.20 (1240)	0.32 (1237)		-	0.43 (1337)
	9	Applied contextual problems	0.42 (1286)	0.19 (1283)	0.29 (1251)	0.20 (1253)	0.37 (1252)	0.49 (1249)			-

All correlations were significant (*p* ≤ 0.001). Numbers of cases are in parentheses.

**Table 6:** Mean and standard deviation of tested variables of the three intellectual ability groups, as well as results of one-way ANOVA and effect size (η^2^).

	Low Intellectual Ability		Average Intellectual Ability		High Intellectual Ability			
	*M*	*SD*	*M*	*SD*	*M*	*SD*	F(*df*, *df*)	η^2^
**t1 (*n*)**	*n* = 135–148		*n* = 910–923		*n* = 135–138			
Age (months) ^1^	64.62	5.06	62.91	4.19	62.15	4.04	10.84 (2, 236) *	.02
Intelligence ^1^	32.59	4.68	49.29	5.28	65.40	3.47	2371.03 (2, 276) *	.72
Attention ^1^	39.92	8.83	48.36	8.74	54.80	5.63	150.54 (2, 264) *	.16
Phonological Loop ^1^	41.92	9.15	49.44	8.24	54.24	6.41	86.09 (2, 245) *	.12
Visuospatial sketchpad	43.93	9.43	49.76	9.47	55.87	9.93	54.54 (2, 1185) *	.08
Central executive	42.24	8.20	50.31	9.01	57.73	9.91	100.29 (2, 1185) *	.15
Mathematical/numerical precursor skills	37.87	8.19	50.14	8.36	59.88	8.30	237.61 (2, 1178) *	.29
**t2 (*n*)**	*n* = 137–145		*n* = 895–922		*n* = 135–138			
Age (months) ^1^	73.91	5.00	72.39	4.02	71.26	3.80	12.80 (2, 236) *	.02
Intelligence ^1^	32.93	4.83	50.14	5.21	65.33	3.32	2319.51 (2, 276) *	.72
Attention ^1^	40.99	8.93	50.03	8.02	55.11	5.96	123.79 (2, 252) *	.17
Phonological Loop ^1^	39.87	9.53	49.48	8.05	53.78	6.74	97.71 (2, 234) *	.16
Visuospatial sketchpad	44.36	8.24	50.00	9.70	55.81	9.33	50.15 (2, 1169) *	.08
Central executive	40.07	8.14	49.86	8.95	57.16	8.71	131.44 (2, 1166) *	.18
Mathematical/numerical precursor skills	37.69	9.35	49.76	8.54	58.72	8.39	206.06 (2, 1164) *	.26
**t3 (*n*)**	*n* = 68–69		*n* = 708–711		*n* = 123–127			
Age (months)	97.13	4.57	94.80	3.98	93.88	3.76	14.89 (2, 903)*	.03
Addition ^1^	−0.49	0.72	−0.04	0.96	0.45	1.17	23.71 (2, 147)*	.05
Subtraction ^1^	−0.61	0.91	−0.01	1.00	0.32	0.94	22.18 (2, 145)*	.04
Applied contextual problems ^1^	−0.85	0.62	−0.08	0.96	0.71	0.97	94.31 (2, 162) *	.13

Variables of t1 and t2 were reported in T-values. Variables of t3 were reported in z-scores. * *p* ≤ 0.001; ^1^ Significant differences between variances were detected (Levene test of homogeneity). In these cases, the more robust Welch’s F was calculated, and Welch’s F and adjusted degrees of freedom were reported instead.

## Data Availability

The data presented in this study are available on request from N.P.
